# Retrograde ejaculation following open ureteric reimplantation: a case report

**DOI:** 10.4076/1752-1947-3-7410

**Published:** 2009-08-18

**Authors:** Eleanor Au, Ranan Dasgupta, Prokar Dasgupta

**Affiliations:** 1 Department of Urology, Guy's Hospital, London, SE1 9RT, UK

## Abstract

**Introduction:**

Retrograde ejaculation is not a recognized complication of ureteric reimplantation surgery. We describe this unusual complication in a 25-year-old man, with no other cause for his ejaculatory dysfunction.

**Case presentation:**

A 25-year-old Caucasian man presented with left hydronephrosis ascribed to a megaureter. Following open reimplantation of the ureter, the patient developed retrograde ejaculation that did not respond to medical therapy.

**Conclusion:**

The key result reported here is that retrograde ejaculation is a possible complication of open pelvic surgery, for which patients should receive counselling. This is relevant for both urologists and general physicians who consult relatively young men with ejaculatory difficulties.

## Introduction

Retrograde ejaculation refers to the propulsion of semen back into the urinary bladder rather than the usual antegrade flow. In urology, this is seen most commonly after transurethral resection of the prostate, with an incidence of up to 85% [1]. Here, we report an unusual case of retrograde ejaculation after ureteric reimplantation for a megaureter in a healthy 25-year-old man.

## Case presentation

A 25-year-old Caucasian man, who was otherwise fit and well, presented with recurrent intermittent left loin pain. An abdominal computed tomography (CT) scan revealed mild left hydronephrosis and a dilated left ureter down to the level of the bladder, with no evidence of renal tract calculi. A MAG-3 diuretic renogram demonstrated good bilateral drainage but with significant dilatation of the left collecting system and ureter, with the left side contributing 49% of the total function, suggestive of a non-obstructive megaureter.

After initial conservative management with 6-monthly reviews for 3 years, the patient developed frequent urinary tract infections accompanied by haematuria and further left loin pain. His left renal function was shown to have deteriorated from 49% to 35% by dimercaptosuccinic acid (DMSA) scintigraphy imaging over a 6-month period. An open ureteric reimplantation was therefore performed, with excision of the distal ureter, tapering and tunnelling of the ureter using the Leadbetter-Politano procedure. The dissection was difficult, with the mega-ureter being quite adherent, and the operative time was two hours. The histopathological findings were consistent with a left mega-ureter in a state of chronic reflux. The patient made a good functional recovery and did not suffer from any further loin pain. A MAG-3 renogram at the follow-up after 2 months showed an increase in the left kidney function from 35% to 43%.

However, three months postoperatively, the patient reported ejaculatory difficulties, and a spermatozoa-containing post-ejaculatory urine sample confirmed a diagnosis of retrograde ejaculation. Cystoscopy revealed normal bladder neck and outflow. He was unresponsive to alpha-agonists and therefore referred for assisted contraception at his request. After a follow-up of three years, the retrograde ejaculation still persists at the time of writing, while the flank pain remains resolved.

## Discussion

There appears to be no previous report of retrograde ejaculation following reimplantation of a megaureter. The causes of retrograde ejaculation can be broadly divided into anatomical, neurogenic or pharmacological. Anatomical causes can involve the bladder neck, for example by being rendered incompetent by transurethral resection of the prostate or bladder neck incision; the urethra, such as urethral stricture or posterior urethral valves; or the extrinsic sphincter, such as failure to relax. Neurogenic causes can be a result of disease such as multiple sclerosis, or injury such as spinal cord injury, or following surgery due to disruption of the innervations such as retroperitoneal lymph-node dissection, after abdomino-perineal resection, or sympathectomy. Various drugs can also prevent normal ejaculation, such as alpha-antagonists, antidepressants or antipsychotics. Of these, the most plausible explanation for our case is that there was some disruption of the innervation responsible for ejaculation.

The normal physiology of ejaculation consists of two phases: emission and expulsion. Emission involves the peristaltic contraction of the smooth muscles of the seminal vesicles, vas deferens, epididymes and prostate, mediated by sympathetic efferents via the hypogastric nerve, with simultaneous relaxation of the external sphincter and contraction of the bladder neck. The inferior hypogastric plexus is situated retroperitoneally on either side of the rectum, posterior to the seminal vesicles. Expulsion involves the forceful propulsion of semen through the urethra by rhythmic contraction of the bulbospongiosus, mediated by somatomotor supply. In contrast, it is mainly the parasympathetic innervation that is responsible for attaining and maintaining an erection. We speculate that the surgical dissection involved in reimplantation may have disrupted the normal innervation derived from the hypogastric plexus. Alternatively, the compression of these nerves, by a haematoma for instance, could lead to adjacent fibrosis and thus indirect disruption of this innervation. If these events were unnoticed intra-operatively, then subsequent morbidity is a possibility. However, there is bilateral innervation, meaning that isolated unilateral injury seems unlikely to result in ejaculatory disturbance. This may be contrasted with our understanding of this side effect following other pelvic surgery such as cystectomy [2] or total mesorectal excision [3], as well as retroperitoneal lymph-node dissection [4], which tend to involve bilateral extent of dissection.

The treatment of retrograde ejaculation is based on underlying aetiology. Anatomical causes, for instance, after prostate surgery, are rarely curable, and sperm harvesting from the urine should be considered if pregnancy is desired. Pharmacological causes are generally reversible by withdrawing the causative drug(s). Patients with neurological conditions may respond to alpha-agonists, such as ephedrine or 50 mg imipramine, which work by closing the bladder neck and thus promoting antegrade ejaculation. There have been some reports on the role of penile vibratory stimulation for the treatment of ejaculatory difficulties, although most have targeted those with anejaculation, such as in spinal cord injury, rather than retrograde ejaculation.

## Conclusion

In conclusion, we report a previously unrecognized complication of open ureteric reimplantation. The mechanism of this remains unclear, though it is possibly related to a local effect on the pelvic innervation.

## Abbreviations

CT: computed tomography; DMSA: dimercaptosuccinic acid.

## Consent

Written informed consent was obtained from the patient for publication of this case report. A copy of the written consent is available for review by the Editor-in-Chief of this journal.

## Competing interests

The authors declare that they have no competing interests.

## Authors' contributions

EA acquired the data and wrote the major part of the work. RD reviewed the literature and helped write the manuscript. PD performed the surgery and reviewed the manuscript. All authors read and approved the final manuscript.
